# Medical Society of the District of Columbia

**Published:** 1871-01

**Authors:** 


### Medical Society of the District of Columbia.

We notice, in the Washington *Sunday Herald*, an account of the Annual meeting of the Medical Society of the District of Columbia. The noticeable feature of the occasion was Dr. J. M. Toner's address, on accepting the office to which he was elected, as President of the Society.

He gave interesting statistics, which we should be glad to publish, as showing the state of this society, which has attracted so much attention in the country the last year. His entire address was highly creditable, both to Dr. Toner, and the society which elected so intelligent, efficient and worthy a man to its first honors.

